# MRI in childhood Arrhythmogenic Right Ventricular Cardiomyopathy and proposed modification of the Task Force Criteria for children

**DOI:** 10.1186/1532-429X-14-S1-O1

**Published:** 2012-02-01

**Authors:** Lars Grosse-Wortmann, Yousef Etoom, Sindu Govindapillai, Brian McCrindle, Cedric Manlhiot, Shi-Joon Yoo

**Affiliations:** 1 Hospital for Sick Children, Toronto, ON, Canada

## Background

ARVC is a genetically determined cardiomyopathy which typically manifests clinically during the second to fourth decade of life. The diagnosis is made using a scoring system of signs and symptoms, known as the revised task force criteria (rTFC). MRI has recently been shown to be of little added value in the diagnosis of ARVC in adults. Its role in the pediatric age group is unclear. We sought assess the usefulness of magnetic resonance imaging (MRI) in the diagnosis of arrhythmogenic right ventricular cardiomyopathy (ARVC).

## Methods

We retrospectively reviewed the MRI studies of all pediatric patients who were referred to MRI for signs of ARVC between 2005 and 2009. Following exclusion of serial studies in the same patient and those with poor image quality, 145 studies were analyzed for wall motion abnormalities (WMA), fibrofatty infiltration, and right ventricular (RV) volume. A diagnosis of possible, borderline, or definitive ARVC was made on the basis of the rTFC.

## Results

**Figure 1:**
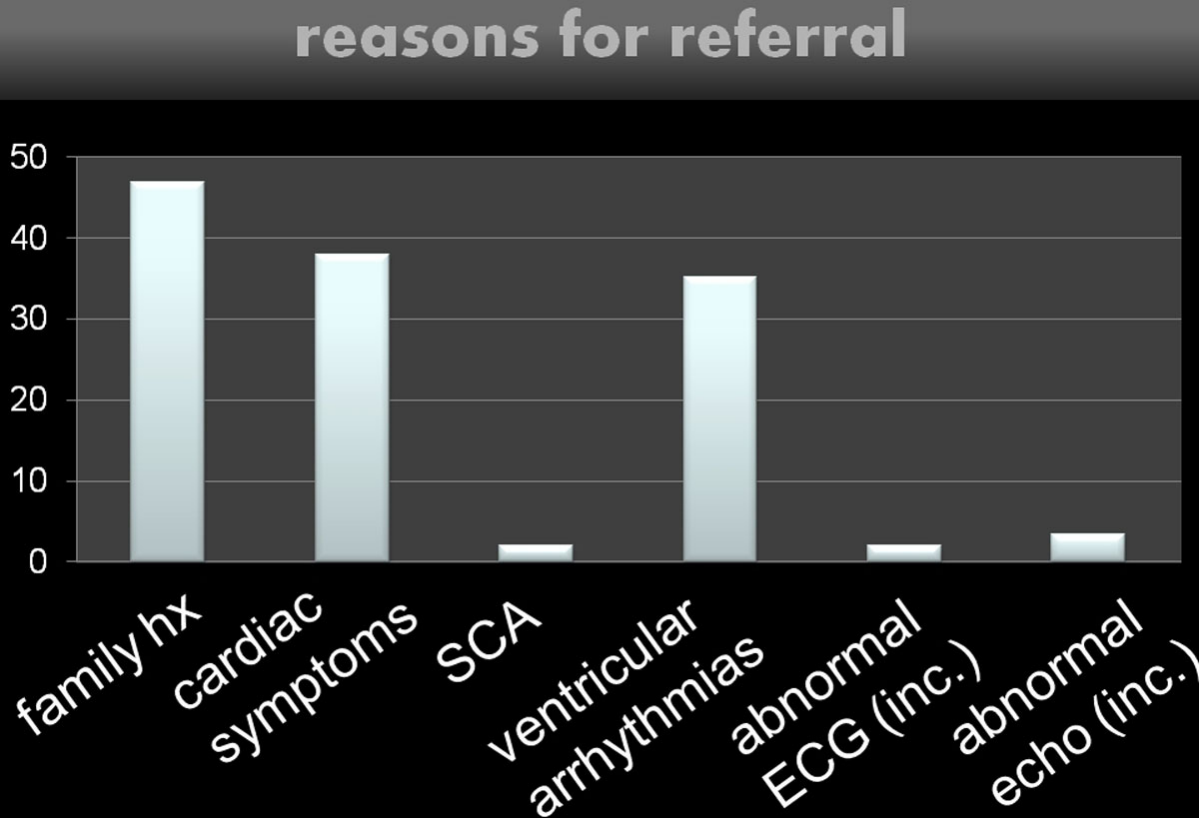
Reasons for referral for MRI to assess for signs of ARVC

**Figure 2:**
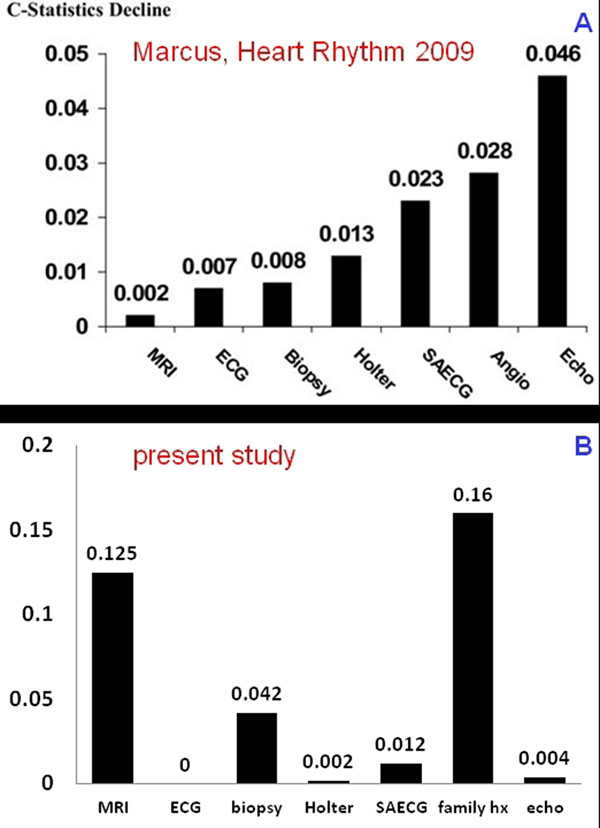
C-Analysis from the paper by (A) Marcus and (B) of our data. The larger the columns the more important the criterion is for the diagnosis of ARVC. MRI and family history are the highest performing criteria in our cohort as opposed to the adult population where echo is most and MRI least important.

Figure 1 shows the reasons for referral. 39% of the patients were unaffected, 27% had possible, 21% borderline, and 13% definitive ARVC. Fatty infiltration and myocardial fibrosis were detected in only 1 and 3 patients, respectively, all of whom had severe WMA. WMA severity correlated with the certainty of the ARVC diagnosis. A c-analysis revealed that the accuracy of the rTFC did not suffer from removing the echo- and electrocardiograms from the diagnostic work-up. On the contrary, removing the family history or the MRI grossly reduced the diagnostic performance of the rTFC. This is in stark contrast to the findings in adults (Figure 2). "Non-rTFC" such as RV thinning, RV outflow tract dilatation, and abnormal trabeculations had a low sensitivity, but high specificity for ARVC. Patients with definitive ARVC had significantly larger left ventricles than those without, possible or borderline ARVC (90ml/m2 vs. 88,89,90ml/m2, respectively).

## Conclusions

Unlike in adults, MRI is a useful and important tool in the diagnostic work-up of ARVC in children and adolescents. The reason lies within the more subtle degree of WMA in this age group which are not detected by echocardiography but found on MRI. In the pediatric age group, fibrofatty degeneration is found rarely and never without wall motion abnormalities. The respective sequences should be omitted in this population. Our data strengthen the concept that ARVC is a global, biventricular disease, rather than an isolated RV cardiomyopathy. Based on our data, we are proposing a modification of the rTFC to exclude certain criteria and incorporate non-rTFC MRI findings, leading to a novel scoring system for pediatric ARVC.

## Funding

No external funding.

